# Fully constrained acetabular liner vs. dual mobility hip joint in the surgical treatment of metastatic bone disease of the hip: study protocol for a randomized, open-label, two-arm, non-inferiority trial evaluating the post-operative hip dislocation rate

**DOI:** 10.1186/s13063-023-07237-9

**Published:** 2023-03-18

**Authors:** Afrim Iljazi, Michala Skovlund Sørensen, Kolja Sebastian Weber, Allan Villadsen, Frank Eriksson, Michael Mørk Petersen

**Affiliations:** 1 Musculosketal Tumor Section, Department of Orthopedic Surgery, Copenhagen University Hospital – Rigshospitalet, Inge Lehmanns Vej 6, 2100 Copenhagen, Denmark; 2 Department of Clinical Medicine, University of Copenhagen, Copenhagen, Denmark; 3 Section of Biostatistics, Department of Public Health, University of Copenhagen, Øster Farimagsgade 5 Opg. B, Building: 15-2-13, Postboks 2099, DK-1014 Copenhagen, Denmark

**Keywords:** Metastatic cancer, Pathologic fracture, Total hip arthroplasty, Constrained liner, Dual mobility

## Abstract

**Background:**

Patients receiving total hip arthroplasty (THA) due to metastatic bone disease of the hip (MBD) are at an increased risk of post-operative joint dislocation compared to other populations. Different joint solutions have been developed with the purpose of reducing the dislocation risk compared to regular THAs. One of these solutions, the constrained liner (CL), has been used increasingly at our department in recent years. This design, however, is prone to polyethylene wear and higher revision rates. An alternative is the dual mobility cup (DM), which has been shown to reduce the risk of dislocation in other high-risk populations. Few studies have investigated DM for THA due to MBD, and no studies have directly compared these two treatments in this population. We therefore decided to conduct a trial to investigate whether DM is non-inferior to CL regarding the post-operative joint dislocation risk in patients receiving THA due to MBD.

**Materials and methods:**

This study is a single-center, randomized, open-label, two-arm, non-inferiority trial. We will include 146 patients with MBD of the hip who are planned for THA at the Department of Orthopedic Surgery, Rigshospitalet. Patients with previous osteosynthesis or endoprosthetic surgery of the afflicted hip, or who are planned to receive partial pelvic reconstruction or total femoral replacement, will be excluded. Patients will be stratified by whether subtrochanteric bone resection will be performed and allocated to either CL or DM in a 1:1 ratio. The primary outcome is the 6 months post-operative joint dislocation rate. Secondary outcomes include overall survival, implant survival, the rate of other surgical- and post-operative complications, and quality of life and functional outcome scores.

**Discussion:**

This study is designed to investigate whether DM is non-inferior to CL regarding the risk of post-operative dislocation in patients receiving THA due to MBD. To our knowledge, this trial is the first of its kind. Knowledge gained from this trial will help guide surgeons in choosing a joint solution that minimizes the risk of dislocation and, ultimately, reduces the need for repeat surgeries in this patient population.

**Trial registration:**

ClinicalTrials.gov Identifier: NCT05461313. Registered on July 15 2022. This trial is reported according to the items in the WHO Trial Registration Data Set (Version 1.3.1).

## Background

The hip is the most frequent surgery-requiring site for metastatic bone disease (MBD) of the extremities [1]. Once disseminated, the metastatic lesions drive an osteolytic cascade, causing pain and affecting limb function [2]. In some patients, pain and local progression of the osteolytic lesion can be managed with radiotherapy or bone conserving treatments such as bisphosphonates [2]. However, some patients experience a progression into a pathologic fracture or an impending fracture despite relevant treatment, while others present with a pathological fracture as the first sign of metastatic disease. In these patients, surgery is warranted with total hip arthroplasty (THA) as the treatment of choice [3].

Total hip arthroplasty is one of the most successful operations within orthopedic surgery and provides many patients with a pain-free and well-functioning hip [4]. According to the Danish Hip Arthroplasty Register, a registry that has collected data since 1995, complications seen after THA are relatively few with the most common causes for all-time implant revision being aseptic loosening and dislocation [5]. A recent Danish register-based study found a 2-year cumulative incidence of dislocations of 3.5% after primary THA due to osteoarthritis, with 75% of dislocations occurring within the first 3 months after surgery [6]. Some populations though, such as patients with MBD, have a higher risk, with hip dislocation rates reported between 7 and 13% [7–9]. A record review of patients treated in our institution showed that 88% of dislocations occurred within the first 6 post-operative months. The dislocation itself is a very painful and devastating experience for the patient and requires hospital readmission with joint reduction performed under general anesthesia in the operating theater. Since the purpose of THA in MBD is not curative but rather to alleviate pain and preserve limb function, and given that the 1-year survival following surgery is approximately 40% [1, 10, 11], it is crucial to select a treatment that minimizes the risk for hospital readmission and avoids repeat surgery. Different articulations have been developed to mitigate the risk of joint dislocation compared to a regular THA. For example, constrained acetabular liners (CL) are used in revision surgeries for patients with repeated dislocations and in other high risk populations [12]. This option has been used increasingly in our department in the past years in patients with MBD. However, constrained liners are prone to polyethylene wear and high revision rates in the long run [13, 14], while the constrained design potentially impedes the patient with a restricted range of joint motion [15]. An alternative to the constrained liner is the dual mobility cup (DM), which has been shown to decrease the risk of dislocation in other high-risk populations such as revision THA patients and hip fracture patients [16–18]. The dual mobility design also provides an added benefit of a less restricted range of motion [19], which potentially could translate to better functional outcomes in everyday life. To date, few studies have evaluated dual mobility cups in patients with MBD, and no study has directly compared these two treatment options in this population. Given that dual mobility cups are proposed to be associated with fewer complications than constrained liners, we want to investigate whether these constitute a viable alternative in patients with MBD. The purpose of this trial is therefore to investigate if dual mobility cups are non-inferior to constrained liners regarding the post-operative dislocation risk in patients with MBD.

## Methods and design

### Study design and objectives

The current study is investigator initiated and designed as a randomized, two-arm, open-label non-inferiority study. The study is a single-center study performed at the Musculoskeletal Tumor Section at the Department of Orthopedic Surgery, Rigshospitalet, Denmark. Patients will be stratified by whether subtrochanteric bone resection is performed and randomly allocated to either CL or DM in a 1:1 ratio. The primary objective is to assess whether DM is non-inferior to CL regarding the post-operative joint dislocation risk. Secondary objectives include the overall survival, implant survival, the post-surgery complication rate, investigator reported outcomes, and patient reported outcomes (see the “ Outcome measures” section).

### Eligibility criteria

We will include patient that meet the following criteria: ≥ 18 years of age, diagnosed with metastatic bone disease of the hip (defined as bone lesions in the proximal femur and/or acetabulum because of a secondary malignant growth of a primary cancer located elsewhere or due to hematological malignancies), determined eligible for THA by a multidisciplinary board of physicians and surgeons. Patients will be excluded if they have previously had an osteosynthesis or endoprosthetic surgery of the ipsilateral hip, are planned to receive partial pelvic reconstruction or total femoral replacement, have previously been enrolled in the study, are unable to provide informed consent, or if it is not surgically viable to insert the acetabular and/or femoral component. In the case of patients referred for bilateral surgery, only the first surgery will be included.

### Enrollment

The Department of Orthopedic Surgery, Rigshospitalet, is a highly specialized tertiary center that maintains one of two dedicated units for orthopedic oncology in Denmark. The musculoskeletal tumor section at Rigshospitalet thus services an intake area that covers three of Denmark’s five regions (Capital Region and Regions of Zealand and Southern Denmark). Patients will be recruited among patients referred to the clinic for surgical treatment of MBD of the hip. Upon receiving the referral, a multidisciplinary board of physicians and surgeons review each case and assess whether surgical intervention is warranted. Subsequently, patients will be recruited among all patients approved for THA due to MBD of the hip. Following approval, a research staff will screen patients for eligibility. Upon arrival to the study site, eligible patients will be informed about the existence of the project by the treating staff. The treating staff will ask if the patient is interested in further information from the research staff. If verbal consent is obtained, a person from the research staff will reach out to the patient and provide thorough oral and written information. An adequate reflection period will be provided before written consent is obtained and any study related procedures are commenced.

### Randomization

Subtrochanteric bone resection is often required for the surgical treatment of metastases around or below the greater trochanter. Patients with subtrochanteric resections are at an increased risk of dislocation because muscles important for joint stability are attached to the greater trochanter and the trochanteric region. Patients will therefore be stratified by whether bone resection below the lesser trochanter will be performed and afterwards allocated to one of two treatment (CL or DM) in a 1:1 ratio. The randomization will be performed using a verified computerized irreversible application—the Research Electronic Data Capture (REDCap). The randomization sequence will be generated using statistical software (*blockrand* function from the R package “blockrand” version 1.5) using permuted blocks with 6–10 subjects per block. The randomization sequence was generated and uploaded to REDCap by AI. Patients will be allocated on the day of the surgery right after the patient has been intubated as part of the surgery-related anesthesia. Treatment allocation will be recorded in the patient record and in the electronic data capture system.

### Surgical procedure

All patients in this study will undergo THA with the insertion of an artificial hip joint due to MBD. All surgeries will be performed by attending surgeons specialized in orthopedic surgery with experience with orthopedic oncology, ensuring an adequate skillset and routine in the procedures. All surgeries will be performed using the posterior approach and with cementation of both the acetabular and femoral components. The acetabular components in this study will either be a constrained liner or a dual mobility cup. The constrained liner used in this study will either be a Freedom Constrained Acetabular Liner or G7 Freedom Constrained Acetabular Liner (Zimmer Biomet). The dual mobility cup used in this study will be the Avantage™ Acetabular System (Zimmer Biomet).

### Follow-up and end of study

Follow-up will be performed on post-operative months 3, 6, 12, and 24. The follow-ups will include a record review of the electronic patient record, an evaluation at the outpatient clinic, and patient-reported questionnaires. The following variables will be obtained and stored in a REDCap database:

- Date of primary completion or study withdrawal
- Reason for study withdrawal (if relevant)
- Implant data (type, femoral head diameter, stem diameter, stem length)
- Surgical data (“knife to skin” time, blood loss)
- Post-operative complications (type, date, action taken)
- Length of hospital stay (days)
- Joint dislocation (date, action taken)
- Complications other than joint dislocation (type, date, action taken)
- Hospital readmission (reason, date)
- Karnofsky Performance Status Score [20] after 3, 6, 12, and 24 months post-operatively
- Musculoskeletal Tumor Society Score (MSTS) [21] after 3, 6, 12, and 24 months post-operatively
- Harris Hip Score (HHS) [22] after 3, 6, 12, and 24 months post-operatively
- European Quality of Life—5 Dimensions Questionnaire (Eq-5d) [23] score after 3, 6, 12, and 24 months post-operatively
- Toronto Extremities Salvage Score (TESS) [24] after 3, 6, 12, and 24 months post-operatively

### Study withdrawal and replacement

Patients have the right to withdraw from the study at any time and for any reason without prejudice to their future medical care by the physician or at the institution. Patients can decline to continue in any protocol-required procedures at any time during the study. Furthermore, the investigator can decide to withdraw a patient from the study at any time prior to study completion for the reasons listed below:

- In-surgery decision to either diverge from the allocated treatment, perform partial pelvic reconstruction, or insert a total femoral replacement
- Loss to follow-up or migration
- Revision surgery of the ipsilateral hip

Patient data up to the withdrawal of the patient will be included in the study. We will record the date and reason for withdrawal. Where permitted, available data can be included after withdrawal of consent. We do not expect loss to follow-up regarding the primary outcome due to the organization of the Danish healthcare system. Healthcare in Denmark is public and taxpayer funded. All hospital contacts are documented in the electronic patient record and stored in the digital health record (Danish: *Sundhedsjournalen*). As hip dislocations are always treated in a hospital setting in Denmark, we expect this information to be available for all patients. In case that a patient is withdrawn prior to completion of the surgery (i.e. before wound-closure is completed), a new patient will be enrolled and randomized until the study includes a minimum of 73 patients that complete the surgery in each of the treatment arms (see the “ Sample size calculation” section).

### Outcome measures

#### Primary outcome

- The 6-months hip dislocation rate in patients receiving a dual mobility cup compared to patients receiving a constrained liner for the surgical treatment of MBD

#### Secondary outcomes

- The 3-month, 1-year, and 2-year hip dislocation rate in patients receiving a dual mobility cup compared to patients receiving a constrained liner for the surgical treatment of MBD
- The 3-months 6-month, 1-year, and 2-year implant survival in patients receiving a dual mobility cup compared to patients receiving a constrained liner for the surgical treatment of MBD
- The 3-month, 6-month, 1-year, and 2-year overall survival in patients receiving a dual mobility cup compared to patients receiving a constrained liner for the surgical treatment of MBD
- The incidence of post-surgical and prosthesis-related complications in patients receiving a dual mobility cup compared to patients receiving a constrained liner for the surgical treatment of MBD. These include but are not limited to deep venous thrombosis, pulmonary embolism, wound infections, periprosthetic infections, periprosthetic fractures, etc.
- The 3-month, 6-month, 1-year, and 2-year Karnofsky Performance Status Score in patients receiving a dual mobility cup compared to patients receiving a constrained liner for the surgical treatment of MBD and that are alive at the defined time point
- The 3-month, 6-month, 1-year, and 2-year MSTS in patients receiving a dual mobility cup compared to patients receiving a constrained liner for the surgical treatment of MBD and that are alive at the defined time point
- The 3-month, 6-month, 1-year, and 2-year HHS in patients receiving a dual mobility cup compared to patients receiving a constrained liner for the surgical treatment of MBD and that are alive at the defined time point
- The 3-month, 6-month, 1-year, and 2-year Eq-5d score in patients receiving a dual mobility cup compared to patients receiving a constrained liner for the surgical treatment of MBD and that are alive at the defined time point
- The 3-month, 6-month, 1-year, and 2-year TESS in patients receiving a dual mobility cup compared to patients receiving a constrained liner for the surgical treatment of MBD and that are alive at the defined time point

### Sample size calculation

The trial is designed with regards to the primary outcome. The sample size calculation is based on a pilot study performed at our department that included 18 patients with constrained Lubinus cups (anti-dislocation ring) and 174 patients with uncontrained Lubinus cups [25]. All patients received a THA due to a pathologic fracture or an impending pathologic fracture because of MBD. The patients with a constrained Lubinus cup had a 1-year dislocation rate at 0%, while those with an unconstrained Lubinus cup had a 7% 1-year dislocation rate. Based on this, we decided on a non-inferiority margin with a 6% absolute risk-difference, as dislocation rate outside of this margin would place DM in the same range as unconstrained THA, which is considered inferior to CL and DM. We expect that the probability of avoiding hip dislocation within 6 months is 99% for CL and 98% for DM. With power at 80% for a one-sided 95% confidence interval, we calculate that a minimum of 73 patients is required in each treatment group for a binary-outcome non-inferiority trial. We will therefore enroll a minimum of 73 patients in each treatment arm amounting to minimum of 146 patients in total.

### Data analysis

Data analysis will be performed both per protocol and on an intention to treat basis. Data analysis will be performed blinded to treatment allocation. We will analyze non-inferiority with the fixed-margin method. We will use a pre-defined non-inferiority margin with a 6% absolute risk difference in this trial. We will conclude non-inferiority of DM compared to CL if the estimated one-sided 95% confidence interval of the difference in cumulative incidence lies entirely below this margin.

## Discussion

To our knowledge, this study is the first randomized trial to compare DM and CL in patients with MBD. Patients with MBD of the hip have an increased risk for adverse events and post-surgical complications. The current study aims to improve our knowledge in selecting the right joint solution for this vulnerable patient-population.

### Risk assessment and ethical considerations

THA is a well-established and commonly performed procedure, performed in thousands of patients each year in Denmark. Both acetabular solutions in this study are currently in use in our department but are currently selected at the discretion of the operating surgeon. THA, as with any other surgery, is associated with the risk of bleeding, wound infections, deep venous thrombosis, neurovascular injury, or death. Furthermore, there is also the risk of THA-related complications such as hip dislocation, limb-length discrepancy, and aseptic loosening of the inserted implant. However, all subjects included in this study have been offered surgery after a careful review by an interdisciplinary board of specialists that has determined that the benefits from surgery outweigh the risks. All surgeries will be performed at a highly specialized tertiary center, by orthopedic surgeons experienced with orthopedic oncology and the specific prosthesis used in this study. The current study is approved by the Scientific Ethical Committee of the Capital Region of Denmark (H-21078128), the Danish Data Protection Agency (P-2022–124), and has been registered at ClinicalTrials.gov (NCT05461313). All patients will receive thorough oral and written information about the project, and written informed consent will be obtained before the commencement of any study-related procedures. The patients will furthermore be informed that participation is voluntary, that they can freely decline study participation, and that they have the right to withdraw from the study at any time and for any reason without prejudice to current or future medical care at the institution.

### Clinical implications and future directions

This study is the first randomized trial to investigate how the type of articulating surface affects the post-operative joint-dislocation rate in patients with MBD, which are considered a high-risk population regarding post-operative dislocation, while also providing important evidence on the safety and post-operative complication rate in patients with MDB who receive a THA. Future studies should investigate which of CL or DM is superior if non-inferiority is concluded. Furthermore, future randomized trials are needed to determine whether certain articulating bearings should be preferred in patients with subtrochanteric resections compared to patients without subtrochanteric resections or in patients with isolated femoral metastases compared to patients who also have acetabular involvement.

## Trial status

All relevant regulatory approvals have been obtained. The trial is ongoing. The first subject was recruited on September 20, 2022.

## Data Availability

Only study investigators declared in this protocol will have access to the data from this trial. Access can be granted to other researchers in an anonymized form upon a reasonable request.

## Abbreviations

CL: Constrained acetabular liner
DM: Dual mobility cup/dual mobility liner
Eq-5d: European quality of life—5 dimensions questionnaire
HHS: Harris hip score
MBD: Metastatic bone disease
MSTS: Musculoskeletal tumor society score
TESS: Toronto Extremities Salvage Score for the lower extremity
THA: Total hip arthroplasty
